# Hemobilia after laparoscopic cholecystectomy that was successfully treated conservatively: Case report

**DOI:** 10.1016/j.ijscr.2020.11.015

**Published:** 2020-11-08

**Authors:** Ryosuke Arata, Senichiro Yanagawa, Yasushi Miyata, Tomokazu Ishitobi, Shinya Kodama, Kazuo Sumimoto

**Affiliations:** a Department of Surgery, Yoshida General Hospital, Japan; b Department of Internal Medicine, Yoshida General Hospital, Japan

**Keywords:** LC, laparoscopic cholecystectomy, CT, computed tomography, CBD, common bile duct, ERCP, endoscopic retrograde cholangiopancreatography, ENBD, endoscopic nasobiliary drainage tube, ERBD, endoscopic retrograde biliary drainage, Case report, Haemobilia, Laparoscopic cholecystectomy, Pseudoaneurysm

## Abstract

**Introduction:**

Hemobilia due to pseudoaneurysm rupture is a rare, life-threatening complication of laparoscopic cholecystectomy (LC) that can cause rapid hemodynamic instability. Therefore, symptoms of hemobilia must be assessed carefully.

**Presentation of case:**

An 88-year-old woman underwent LC in our hospital, and blood tests revealed elevation of hepatobiliary enzyme levels on postoperative day (POD) 12. Computed tomography (CT) showed a high absorption area in the common bile duct (CBD), and a diagnosis of hemobilia and a pseudoaneurysm without active bleeding into the abdominal cavity was made. There was no leakage of contrast medium outside the CBD during endoscopic retrograde cholangiography; thus, an endoscopic nasobiliary drainage (ENBD) tube was inserted on POD 12 and an endoscopic retrograde biliary drainage (ERBD) stent was placed in the CBD on POD 13. Thereafter, hepatobiliary enzyme levels gradually normalized and the ENBD tube and ERBD stent were removed on POD 27 and POD 54, respectively. The patient was discharged on POD 66.

**Discussion:**

Hemostasis establishment using surgery or intervention radiology is often required for the treatment of hemobilia due to pseudoaneurysms; however, conservative treatment was effective in our case and we were able to pursue a minimally invasive approach. Erosion due to clip penetration or incomplete clipping of the cystic artery or its branches during surgery may have caused the cystic artery pseudoaneurysm.

**Conclusion:**

Hemobilia could be life threatening and cause acute hemodynamic instability; therefore, prompt diagnosis is required. Although the frequency of complication is low, the possibility of hemobilia after LC should be considered.

## Highlights

• Hemobilia from pseudoaneurysm rupture after laparoscopic cholecystectomy is rare.
• Due to consequent rapid hemodynamic instability, it could be life threatening.
• Non-contrast CT and ERCP examinations were performed to confirm hemobilia diagnosis.
• Investigations revealed that there was no contrast agent leakage.
• Thus, our patient was treated conservatively as opposed to surgically.

## 1 Introduction

Hemobilia was first defined by Sandblomm in 1948 as a hemorrhage in the biliary tract resulting from an abnormal communication between blood vessels and bile ducts [1]. Severe right upper quadrant pain, hematemesis or melena, and elevated bilirubin are the three signs of hemobilia, according to Grove et al. [2]. In a study of 222 cases, Green et al. [3] reported that the causes of hemobilia were iatrogenic (65%), inflammation (13%), tumors (7%), trauma (6%), and others (9%); therefore, iatrogenic cause is the most important factor. Additionally, hemobilia has been reported as a rare complication of laparoscopic cholecystectomy (LC) due to the formation of pseudoaneurysms, and hemostasis through surgery or radiological intervention is often required [4].

Herein, we report a case of hemobilia after LC that was successfully treated conservatively with an endoscopic nasobiliary drainage (ENBD) tube and endoscopic retrograde biliary drainage (ERBD) stent. There are no reports on a conservative treatment for hemobilia after LC. Therefore, we believe that our report provides evidence that supports conservative treatment as a possible alternative to surgical interventions in some cases.

This work has been reported in line with the SCARE criteria [5].

## 2 Presentation of case

An 88-year-old woman with complaints of vomiting was brought to our hospital in an ambulance. She had a history of diabetes mellitus, paroxysmal atrial fibrillation, and chronic renal failure with an estimated glomerular filtration rate (eGFR) of 25 mL/min/1.73 m^2^. She was diagnosed with acute cholecystitis and underwent LC, which was performed by a gastroenterologist. Her gallbladder was severely inflamed, but the cystic duct and the cystic artery were both identified and dissected after being double clipped; the entire surgery was uneventful.

**Fig. 1:**
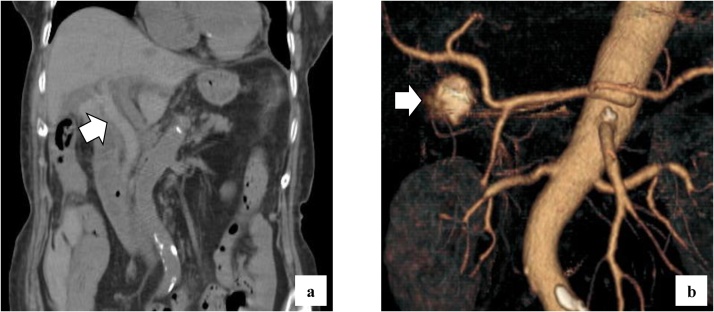
CT findings CT reveals a high absorption area in the CBD (a) and a 20-mm aneurysm in the cystic artery.

**Fig. 2:**
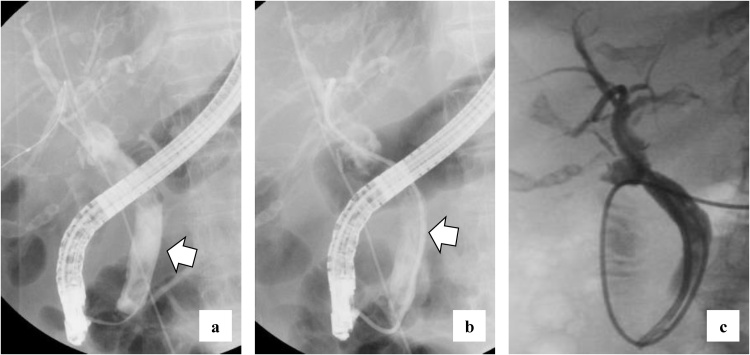
ERCP findings There is no leakage of contrast medium outside the CBD (a) and the ENBD tube was placed on POD 12 (b). Contrast examination using the ENBD tube show no leakage of contrast medium outside the CBD on POD 27 (c).

She complained of back pain and right hypogastric pain on postoperative day (POD) 12. Blood examination revealed anemia and elevated levels of serum bilirubin and hepatobiliary enzymes. The total bilirubin (T-bil) level was 2.73 mg/dL (normal range: 0.2–1.2 mg/dL), direct-bilirubin (D-bil) level was 2.36 mg/dL (normal range: 0–0.4 mg/dL), aspartate aminotransferase (AST) level was 243 U/L (normal range: 10–40 U/), alanine aminotransferase (ALT) level was 82 U/L (normal range: 5–40 U/L), alkaline phosphatase (ALP) level was 2018 U/L (normal range: 100–340 U/L), and hemoglobin (Hb) level was 6.5 g/dL (normal range: 12–15 g/dL). Computed tomography (CT) revealed a high absorption area in the common bile duct (CBD), and hemobilia was suspected (Fig. 1a). Contrast-enhanced CT revealed no active bleeding into the abdominal cavity, but a pseudoaneurysm was observed in the cystic artery (Fig. 1b). Endoscopic retrograde cholangiopancreatography (ERCP) revealed contrast deficiency in the CBD, although there was no leakage of contrast outside the CBD (Fig. 2a). An ENBD tube was inserted on POD 12 (Fig. 2b) and an ERBD stent was placed in the CBD on POD 13. The fluid drained from the ENBD tube was a mixture of blood, bile, and pus, and was suggestive of a superimposed bacterial infection. A diagnosis of hemobilia was made as the fluid drained from the ENBD was blood. The patient also presented with melena. Therefore, conservative treatments, such as blood transfusion and administration of antibiotics, were commenced. Due to gradual deterioration of renal function (eGFR reduced to 7 mL/min/1.73 m^2^) and reduction in urine output during the treatment, hemodialysis was temporarily introduced on POD 15. After controlling hemobilia and biliary infection, renal function improved (eGFR increased to 23 mL/min/1.73 m^2^) and hemodialysis was discontinued on POD 30. Blood-based investigations revealed that the levels of hepatobiliary enzymes were improving gradually, while contrast examination using the ENBD tube showed no leakage of the contrast medium outside the CBD on POD 27 (Fig. 2c). The ENBD tube was removed on POD 27, the ERBD stent was removed on POD 54, and she was discharged on POD 66.

**Fig. 3:**
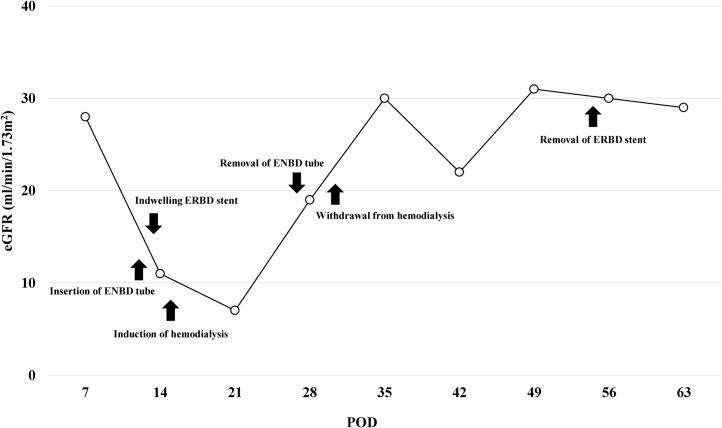
The transition of eGFR and the clinical course of the patient from POD 7 to POD 63.

The clinical course of the patient from POD 7 to POD 63 is shown in Fig. 3.

After discharge, an outpatient follow-up was performed once a month and the follow-up period was of 46 days. Blood investigations performed on POD 112 revealed that the hepatobiliary enzymes were within their normal ranges (T-bil: 0.57 mg/dL, D-bil: 0.27 mg/dL, AST: 29 U/L, ALT: 18 U/L, and ALP 327 U/L) and the renal function was no longer impaired (eGFR: 31 mL/min/1.73 m^2^).

## 3 Discussion

Currently, a majority of cholecystectomies are performed laparoscopically, which is associated with a decrease in abdominal wall trauma and earlier patient mobilization and discharge [6]. However, although medical technology has been advancing, complications occur at a constant rate. Although very rare, pseudoaneurysms of the hepatic or cystic artery are serious complications of LC. The mechanism of pseudoaneurysm formation after LC is still unclear, but it has been reported that direct vascular injury, thermal injury, erosion due to clip intrusion, bile leakage, and infection are possible etiologies [7]. Additionally, rupture of a pseudoaneurysm can lead to an abnormal communication between an artery and a bile duct, and result in hemobilia. A majority of pseudoaneurysms appear within 6 weeks after LC [8,9], although there are case reports on pseudoaneurysms that appeared over 20 months after LC [10]. Pseudoaneurysm formation is difficult to determine, as asymptomatic aneurysms cannot be easily detected and they may thrombose spontaneously. The risk of rupture is also dependent on the size of the pseudoaneurysm [11]. Hemobilia due to pseudoaneurysms is a rare, but potentially life-threatening complication; therefore, appropriate diagnosis and treatment is essential.

Wen et al. reported that abdominal CT was useful for the diagnosis of hemobilia and also reported 14 cases of hemobilia after LC, all of which were treated with interventional radiology (IVR) [12]. IVR techniques, such as transarterial embolization, are generally selected as the first choice due to their high success rates [8,9,13]. If anemia progresses or the patient is hemodynamically unstable, selective angiography may be useful with or without the leakage of contrast on CT. However, contrast-enhanced CT or IVR techniques require a contrast medium, which may cause renal dysfunction [14]. Therefore, caution is required when using contrast, especially in patients with renal failure [15]. There are reports on anatomical variations of the cystic artery in a literature review of over 9800 cases. According to this review, multiple cystic arteries were observed in 8.9% of the cases [16]. Therefore, if possible, it is beneficial to perform contrast-enhanced CT before surgery to detect these anatomical variations.

In our case, hemobilia could have been caused by erosion due to clip penetration of the cystic artery or from a branch of cystic artery that was not detected during surgery. However, there was no obvious damage to the cystic duct, CBD, or cystic artery during the operation. We did not choose any IVR technique and decided to manage the patient conservatively with ENBD and ERBD, because contrast-enhanced CT revealed no active bleeding into the abdominal cavity and she was hemodynamically stable. In addition, we were able to avoid a permanent requirement of hemodialysis due to deterioration of renal function secondary to contrast medium usage.

Currently, there is no consensus on the management of hemobilia after LC; however, conservative treatment may be the best option if the patient’s condition is optimum.

## 4 Conclusion

We reported a case of hemobilia in a patient with a pseudoaneurysm after LC that was treated conservatively. Although the frequency of complication is low, the possibility of hemobilia development after LC should be considered, the related signs and symptoms should be recognized promptly, and the necessary treatment should be initiated. There are no reports of conservative treatment for hemobilia after LC. In some cases, less invasive conservative treatment may be possible. However, we should not hesitate to perform surgery or IVR according to the patient`s condition.

## Declaration of Competing Interest

The authors have no conflicts of interest.

## Funding

This research did not receive any specific grant from funding agencies in the public, commercial, or not-for-profit sectors.

## Ethical approval

Ethical approval was not required and patient identifying knowledge was not presented in the report.

## Consent

Written informed consent has been obtained from the patient for the publication of this case report and any accompanying images.

## Author contribution

RA and SY participated in treatment of the patient, collected case details, literature search and draft the manuscript. YH, NK, HT and KS participated in treatment planning of the patient. YM and TI participated in treatment of the patient. SK participated in treatment planning of the patient and helped to draft the manuscript. All authors read and approved the final manuscript.

## Registration of research studies

Not applicable.

## Guarantor

Senishiro Yanagawa.

## Provenance and peer revaiew

Not commissioned, externally peer-reviewed.
